# ALDH Expression in Angiosarcoma of the Lung: A Potential Marker of Aggressiveness?

**DOI:** 10.3389/fmed.2020.544158

**Published:** 2020-10-30

**Authors:** Beatrice Aramini, Valentina Masciale, Daniel Bianchi, Beatrice Manfredini, Federico Banchelli, Roberto D'Amico, Federica Bertolini, Massimo Dominici, Uliano Morandi, Antonino Maiorana

**Affiliations:** ^1^Division of Thoracic Surgery, Department of Medical and Surgical Sciences, University of Modena and Reggio Emilia, Modena, Italy; ^2^Department of Medical and Surgical Sciences, Center of Statistic, University of Modena and Reggio Emilia, Modena, Italy; ^3^Division of Oncology, Department of Medical and Surgical Sciences, University of Modena and Reggio Emilia, Modena, Italy; ^4^Department of Medical and Surgical Sciences, Institute of Pathology, University of Modena and Reggio Emilia, Modena, Italy

**Keywords:** target therapy, aldehyde dehydrogenase (ALDH), malignant rare lung tumor, rare tumor, angiosarcoma, marker, angiogenetic process, malignant vascular tumors

## Abstract

**Background:** Primary angiosarcoma of the lung is a very aggressive rare malignant disease resulting in a severe prognosis (1). This type of cancer represents about 2% of all soft tissue sarcomas and has a high rate of metastasis through the hematogenous route. For the rarity of this malignant vascular tumor it is still challenging to set a diagnosis (1). The diagnostic features that have thus far been considered include primarily clinical and radiological findings. In some cases, immunohistochemical characteristics based on the most common markers used in pathology have been described. The aim of this report is to present two cases of angiosarcoma of the lung in which the aldehyde dehydrogenase (ALDH) marker was analyzed by immunohistochemistry.

**Methods:** We report two cases of angiosarcoma of the lung in patients underwent lung surgery at our Unit. In addition to the standard histopathological analysis for this disease, immunohistochemistry using an ALDH1A1 antibody was performed in both of the cases. For ALDH quantification, a semi-quantitative method based on the positivity of the tumor cells was used: 0 (<5%), 1 (5–25%), 2 (>25–50%), 3 (>50–75%), 4 (>75%).

**Results:** One patient with recurrent lung disease survived, achieving complete remission after chemo- and radiotherapy. The second patient died of recurrent disease within 5 years of diagnosis. ALDH1A1 was evaluated in both of these cases using an immunohistochemistry scoring system based on the positivity for this marker. The scores were consistent with the patients' clinical outcomes, as the lower (score 1) was observed in the patient with the better clinical outcome, while the higher (score 3) was seen in the patient with the worse outcome.

**Conclusion:** Our data suggest that ALDH may be an important clinical marker in angiosarcoma of the lung. Although further studies need to be performed in a larger cohort of patients, we believe that, if the results will be confirmed, ALDH1A1 may be used to stratify patients in terms of prognosis and for targeted therapy.

## Background

Angiosarcoma is a rare form of malignant vascular tumor occurring for the 2% of soft tissue sarcoma (1–3). Clinical symptoms are similar to those observed in other pulmonary tumors (2). The radiological presentation is highly variable, ranging from a solitary nodule to multiple bilateral lesions. In addition, due to its clinicopathological similarity to metastatic angiosarcoma of the lung, a diagnosis of primary disease requires a complete clinical and radiological examination of the body in order to ensure that there are no primary lesions outside of the chest. Therefore, immunohistochemistry is the gold standard technique to make a diagnosis of angiosarcoma of the lung; in particular, the most used markers are Factor VIII, CD31, CD34, and Fli-1 (4). There is not yet a standardized therapy for this disease; however, therapy usually consists of surgical treatment, chemotherapy, and radiotherapy, depending on the clinical presentation and the number of lesions.

However, aldehyde dehydrogenase (ALDH) which is an enzyme able to detect normal and malignant stem/progenitor cells (5) is now used as marker in tumor initiating cells (TICs) especially for the two ALDH isoforms, ALDH1A1 and ALDH1A3 (6–8). In particular, ALDH1A1 has been considered the most effective markers in consideration of poor prognosis for lung cancer and it is considered a marker of stemness, promoting angiogenesis, which is one of the main characteristics in high vascularised tumor as the angiosarcoma (9–11). Moreover, ALDH1A3, which is another ALDH isoform, is mainly enrolled in the regulation of matricellular proteins promoting the vascular smooth muscle cell proliferation (12).

ALDH has also the capacity to regulate the most important cells functions as self-renewal, expansion, differentiation and drug resistance (13). A deeper knowledge of the ALDH molecular pathways will be useful in many specialties, as regenerative medicine and oncology. In particular, a current clinical trial demonstrates the importance of comparing the effects of the ALDH inhibitor dimethyl ampal thiolester (DIMATE) on normal and malignant hematopoietic cells and stem cells (9, 13). In particular, it has been shown that DIMATE could potentially be used to selectively eliminate cancer cells (9). Although these results were obtained in hematological disease, they demonstrated the importance of ALDH in oncology and suggested that ALDH may be considered not only a marker, but also a target.

In this report, we present our experience with two cases of primary angiosarcoma of the lung that had completely different clinical presentations and, therefore, completely different treatments. In particular, we found that, while both of the angiosarcomas showed ALDH expression, high and low ALDH scores were associated with worse and better prognosis, respectively. Further studies on the presence of this marker in angiosarcomas of the lung may highlight the importance of ALDH as a prognostic factor and as a potential biomarker for targeted therapies.

## Methods

### Case 1

**Figure 1:**
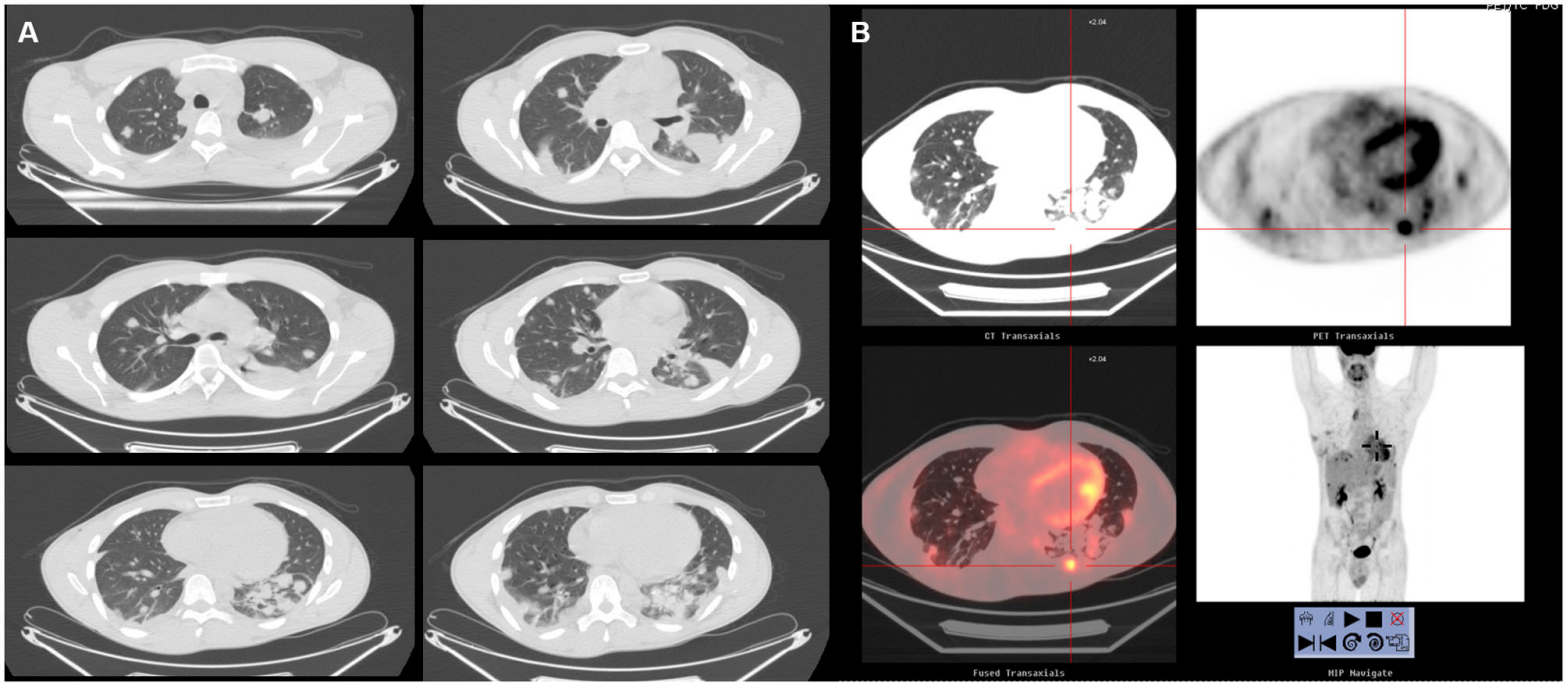
**(A)** Chest CT in case 1 before treatment. The lungs showed bilateral nodules. **(B)** Total body ^18^F-FDG PET. No hypermetabolic lesions other than the lesions in the lungs.

A 27-year-old man arrived at our Department in June 2016 after multiple episodes of hemoptysis followed by intense chest pain. He referred only previous episodes of inflammatory bowel disease (IBD). Chest X-rays showed multiple bilateral lesions in the lungs that were confirmed by the computed tomography (CT) scan in which multiple pseudonodular features surrounded by a ground glass halo were identified in both lungs (Figure 1A). The abdomen and brain CT scan were negative for atypical lesions. A PET scan was performed, revealing evidence of multiple active sites in the lungs (Figure 1B). In June 2016, transbronchial biopsies were non-diagnostic and the patient underwent surgical left lung biopsies performed using a video-assisted thoracic surgery (VATS) approach. The pathological exam revealed spindle cells of endothelial origin that stained positive for CD31 and partially positive for cytokeratin AE3/AE4. Staining for CD34, TTF1, and CD30 were found to be negative. An angiosarcoma of the lung was diagnosed.

**Figure 2:**
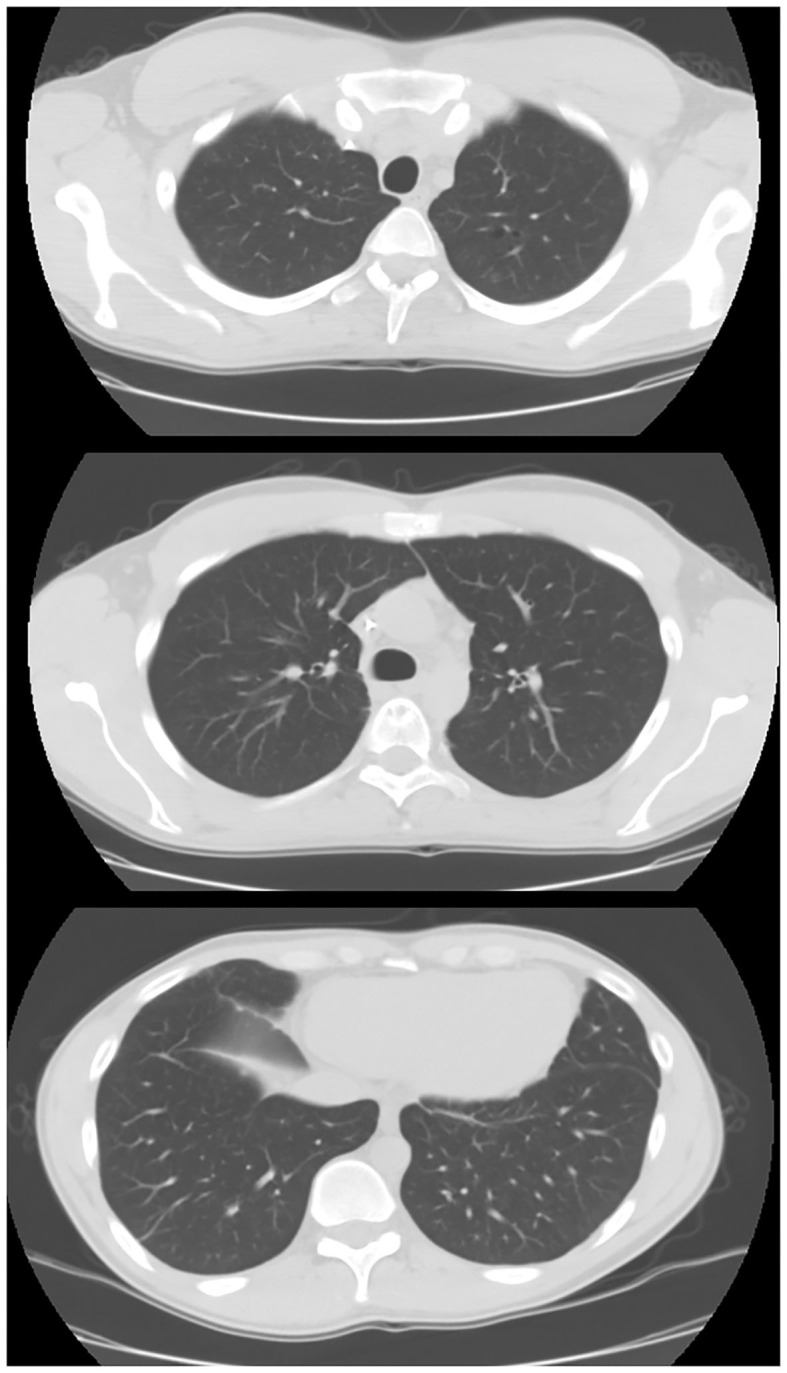
Chest CT in case 1 after chemo- and radiotherapy treatment. After 1 year of treatment, the patient showed a complete remission of the disease.

The patient was sent to the oncologist. A new chest CT performed in July 2016 showed a recurrence at the level of the thoraco-abdominal passage at the cardiophrenic angle and caudally to the right lower lobar pulmonary vein; after positioning of a port-a-cath (PORT device), patient started first-line chemotherapy with epirubicin and ifosfamide. The revaluation after three cycles revealed an excellent response in the parenchymal lung lesions, a net reduction of bilateral pleural effusion, and a reduction in the lesion at the level of the D5 soma in net volumetric evolution. On October 2016, radiosurgery (18 Gy in single dose) on the D5 lesion was performed. Another three cycles with the same scheme followed. In January 2017, due to lung progression, the patient was started on second-line chemotherapy with docetaxel plus gemcitabine associated with radiotherapy until April 2017 (four total cycles given); starting in April, the therapy continued with only gemcitabine. After these treatments, in May 2017, the patient showed additional hemoptysis episodes, which drew attention to a recurrence at the level of the left lower lobe. In August 2017, he started weekly paclitaxel and achieved a complete remission of the disease, which has been confirmed in the subsequent follow-up examinations. The most recent chest CT (performed in January 2020) showed a complete remission of angiosarcoma (Figure 2).

### Case 2

A 61-year-old woman came to our hospital for persistent cough in the last 2 years, initially treated as an allergic bronchiolitis without any improvement. Her medical history was notable only for a prior isterectomy due to fibromatosis. She denied smoking habit.

In August 2005, a chest X-ray showed a small single lesion in the left lung. At the same time, a CT scan of the chest showed evidence of hyper-dense tissue of ~2 cm around the lower left bronchus.

In September 2005, we performed a bronchoscopy with transbronchial biopsy, leading to the diagnosis of angiosarcoma. To ensure that it was not a metastatic lesion, we performed a magnetic resonance imaging (MRI) of the abdomen followed by an 18-fluorodeoxyglucose integrated with computed tomography (^18^F-FDG PET/CT). Neither showed evidence of any other atypical lesion. Primary angiosarcoma of the lung was diagnosed.

In October 2005, a left lower lobectomy was performed by thoracotomy, without any major complications after the surgical procedure.

The histological examination confirmed the initial diagnosis of angiosarcoma, with immunohistochemistry positive for myocyte nuclear factor (MNF116) and CD34 and partially positive for Factor VIII and CD31. A hilar lymph node was found positive for local invasion.

After 1 month from surgery, she was undergone chemotherapy with docetaxel plus gemcitabine (four total cycles given), associated with radiotherapy on the mediastinum (~50 Gy in 25 fractions). Patient performed 3 years follow up by chest and abdomen CT with contrast enhancement until 2008, which showed normal radiological findings, free from the tumor lung disease.

In 2009, the patient was admitted to our Unit for left thoracic pain and suspicious for rib fractures. She underwent to a chest CT with contrast enhancement showing a neoformation of 3.5 × 2 × 1.5 cm at the level of the left intercostal space, between the tenth and the eleventh rib. Due to the previous history of angiosarcoma of the lung, patient underwent surgery in general anesthesia to remove the mass and make a diagnosis. An excision was performed at the level of the chest neoformation with partial removal of the ninth and tenth left rib and a plastic reconstruction of the left thoracic wall. Patient stayed clinically stable and she was discharged from hospital after 6 days without complications. The oncologic team evaluated the patient after few weeks from surgery and a chemotherapy plan was set with only gemcitabine for other 4 months, with no complete remission. The patient died in early 2010 due to insufficient respiratory function caused by lung recurrence.

#### Immunohistochemistry

Paraffin was firstly removed from tissue section with the use of xylene, and subsequently a descending scale of ethyl alcohol (100, 90, 80, and 70°) was used to rehydrate the slices before the washing procedure with phosphate-buffered saline (PBS). The antigen retrieval was performed with a 10 mM sodium citrate buffer ad a high temperature water bath (95°C). After 15 min the slices were washed with PBS for 30 min. To suppress the interference of endogenous peroxidase a solution of 3% H_2_O_2_ was used to dip samples. Therefore, serum was necessary for blocking non-specific signal. At the end of this procedure primary antibodies were added for hours to tissue slices (ab-134188, Abcam, Cambridge, MA, USA), anti-CD34 (Ventana Medical Systems, Arizona, USA), and anti-CD31 (Cell Marque, California, USA). Furthermore, sections were incubated for 30 min with a secondary antibody (PK-4001; Vector Labs, USA) before three times washing with PBS. Samples were subsequently incubated with ABC-HRP (PK-4001; Vector Labs, USA), washed in PBS in order to remove the excess antibody, and stained with 3,3-diaminobenzidine (DAB). Finally, a secondary stain with Mayer's hematoxylin was applied to provide contrast in the section. At the end slices were dehydrated with an ascendant alcohol grade scale (70, 80, 90, and 100°), and then subsequently mounting media was added. Tissue sections were analyzed using a Zeiss Axioskop microscope, with a Zeiss AxioCam ICc 3 High-Resolution Microscope Camera, at 10 × and at 20 × magnification. Blinded a and independent evaluation of the sample were performed to assign the right score for the evaluation of positive cells, in accordance to a previous score (6–8). Immunoreactivity for ALDH marker was assess by a semi-quantitative method based on this range of positivity for the tumor cells: 0 (<5% positive), 1 (5–25%), 2 (>25–50%), 3 (>50–75%), and 4 (>75%) (7).

## Results

### Clinical Results

#### Case 1

After 1 year of chemotherapy and radiotherapy cycles, the patient showed a complete remission of the disease. The last chest CT performed in January 2020 showed complete remission.

#### Case 2

After 5 years of treatments, the patient died in 2010 due to respiratory insufficiency caused by this malignant lung disease.

### Immunohistochemical Evaluation of the ALDH Stem Cell Marker in Two Cases of Angiosarcoma of the Lung

**Figure 3:**
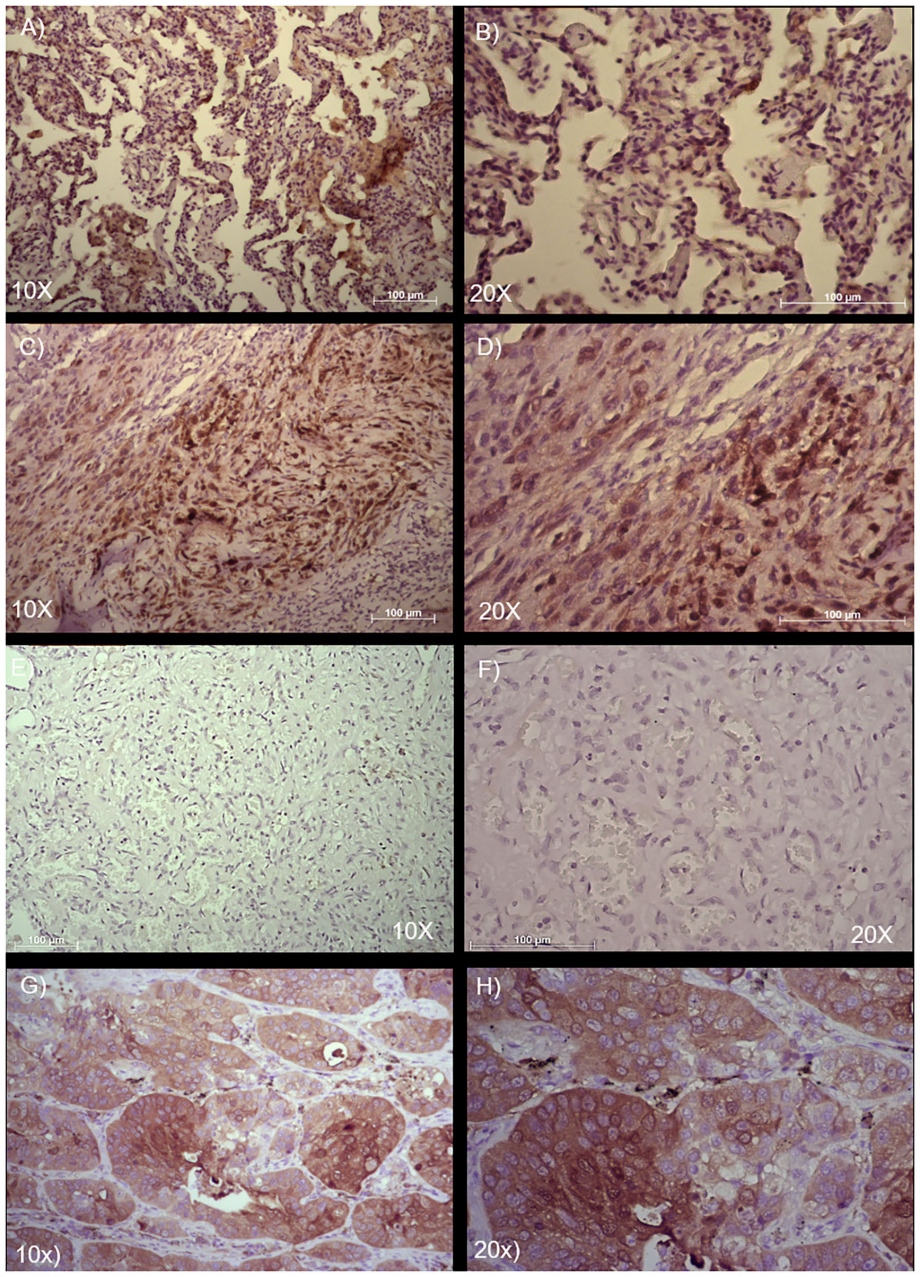
Immunohistochemical analysis and quantification of ALDH-positive cells in two cases of angiosarcoma of the lung. Paraffin-embedded tissues were stained with an ALDH antibody to detect positive cells in these two different cases of angiosarcoma of the lung. **(A,B)** Case 1 and **(C,D)** Case 2. **(E,F)** Isotype control of anti-ALDH antibody. **(G,H)** Adenocarcinoma of the lung used as positive control of anti-ALDH antibody. Semi-quantitative method to measure ALDH based on the positivity of the tumor cells: 0 (<5% positive), 1 (5–25% positive), 2 (>25–50% positive), 3 (>50–75% positive), and 4 (>75% positive). Representative images are shown, with 10 × and 20 × magnification. Scale bar = 100 μm.

The positivity to the ALDH marker was assessed for these pulmonary angiosarcoma cases, using a previous score system (10–12, 14). Samples were examined at 10 × and at 20 × magnification to assign the appropriate score value. In accordance with the literature, in the normal bronchial epithelium ALDH1A1 expression was assed (8). These two patient samples had a different expression of ALDH1A1 staining in the tumor fraction: for case 1, the score was 1 (<5–25% positive tumor cells), while in case 2 the score was 3 (>50–75% positive tumor cells) (Figure 3). The scores seem to be consistent with the clinical outcome of these two patients, as the lower score was observed in the patient with the better clinical outcome, while the higher score was measured in the patient with the worse clinical outcome.

## Discussion

Angiosarcoma of the lung is an extremely rare tumor described in only a few case reports because of its low prevalence in the population. However, it is very interesting to study because of the aggressiveness of this disease which tends to be spread in different part of the body. The rate of recurrence is very high for the intrinsic properties of this tumor with a 5-year survival rate around 20–35% (15–17), which is in contrast to the 5-year survival for all types of soft tissue sarcomas, around 65% (18).

The approaches to treat this malignancy are primarily surgery (in operable patients) and chemoradiotherapy for advanced disease. Table 1 shows an updated information from the recent literature (10–12, 14–18, 20–29), highlighting the clinical and surgical approaches, as well as also the most frequently used markers for this disease.

**Table 1:** Angiosarcoma of the lung.

**References**	**Patients enrolled (n)**	**Gender** **(M/F)**	**Age**	**Type of treatment (surgery and/or medical treatment)**	**Type of resection**	**Type of treatment**	**Immunohistochemistry (IHC) markers**
Aramini et al. (19)	2	1 M 1 F	27 61	Diagnostic surgery + chemotherapy and radiotherapy 2. Surgery + chemotherapy	Surgical left lung biopsies 2. Left lower lobectomy	First line (epirubicin and ifosfamide + radiotherapy); second line (docetaxel plus gemcitabine) and then only gemcitabine 2. Four cycles gemcitabine	CD31 + CD 34 +
Carillo et al. (20)	1	M	56	Chemotherapy	Not resectable for advanced disease.	Adriamicin, II line ifosfamide	AE1/AE3 + CD31 + EMA + Vimentin +++ CD34 +++ (CEA, TTF-1, calretinin, trombomodulin, S100, HMB45, melan-A, Bcl-2, actin, desmin, CD117-)
Chen et al. (21)	2	1 F 1 M	41 50	Surgery + chemotherapy 2. Surgery + chemotherapy	Wedge resection middle lobe 2. Left lower lobectomy and lingula segmentectomy	Sorafenib 2. Four cycles Docetaxel + cisplatin	CD31 + CD 34 + Factor VIII + (CK, EMA, SMA, HMB-45, desmin, S100, ALK-)
Maglaras et al. (22)	1	M	46	Diagnostic surgery + chemotherapy	Right surgical biospy	Adriamicin + ifosfamide	Vimentin + CD31 + (EMA, CEA, S-100, pancytocheratin, cheratin, Leu-M, CD34, Factor VIII, desmin, NSE-)
Modrzewska et al. (23)	1	F	65	Diagnostic surgery	Lung surgical biopsy	/	CD31 + CD34 + (AE1/AE3, calretinin-)
Ozcelik et al. (24)	1	M	62	Surgery	Right upper lobectomy	/	CD31 + CD34 + Factor VIII + CAM 5.2 +
Palvio et al. (25)	1	M	59	Surgery	Right pneumonectomy	/	Factor VIII + Vimentin + (chetarin, desmin, EMA-)
Pandit et al. (26)	1	F	79	Surgery + chemotherapy	Wedge resection Left lower lobe	/	Factor VIII + Vimentin + CD34 +
Ng et al. (14)	1	M	60	Surgery	Left upper lobectomy	/	ERG + CD31 + (CD34, TTF-1-)
Yang et al. (27)	1	M	41	Surgery	Wedge resection left upper lobe	/	Vimentin + CD34 + CD31 + (CK7, CK5, CK20, CK8, S-100, AE1/AE3, TTF-1, Napsin-A, HMB-45, sinaptofisin, CD68, EMA, LCA, actin, desmin-)
Tanaka et al. (28)	1	M	48	Surgery + chemotherapy	Left pneumonectomy	/	ERG + CD31 + Factor VIII + FLI-1 + P53 + (CD34, C2-40, CK7, CK20, EMA, TTF-1, HMB-45, CD10)
Shirey et al. (29)	1	M	65	Surgery + chemotherapy	Wedge resection Right upper lobe	Gemcitabin and Docetaxel	CD31 +++ Vimentin +++ AE1/AE3 + Pancytocheratin + CK7 + EMA + (CAM5.2, CK5/6, p63, S-100, desmin, SMA, Factor VIII, TTF-1, napsin-A, CD34, D2-40, CD21-)
Sheppard et al. (30)	1	M	65	Diagnostic surgery	Lung biopsy	/	CD31 + Factor VIII + CD34 + CAM 5.2 +
Zhang et al. (31)	1	M	72	Surgery for age	Left lower lobectomy	/	CD34 + CD31 + FLI-1 + Vimentin +

*Review of the literature on the surgical and medical approaches in the past and at the present time. The table shows also the markers used in each study to highlight the diagnosis*.

It has been recently showed that ALDH identifies tumor cells with increased affinity for primary tumors, as well as a high capacity to disseminate in many organs (30–32). The scientists have recently found a correlation between ALDH and the capacity to calibrate the tumor cells dissemination into the body (30–33). In particular, different ALDH isoforms seem to be able to regulate the cells diffusion into several solid tumors (30–33). In an interesting study published in 2014 by Nicholas Greco et al. it has been demonstrated a significant correlation of aldehyde dehydrogenase (ALDH) activity and the presence/absence of distant metastases in ten consecutive cases of human bone sarcomas (34). Although angiosarcoma is more difficult to investigate for the rarity of this tumor into the population, it is important to understand the possible role that ALDH may have in this tumor for the possibility to set new target treatment against this aggressive malignant disease. Another interesting aspect recently described which needs to be considered regards the central role of ALDH into the angiogenetic process, detected into the endothelial stem-like cells in vascularized tumors (35, 36). This aspect may strongly confirm the possible presence of ALDH even in a vascularized tumor as the angiosarcoma of the lung.

In our report, we showed two cases of angiosarcoma of the lung with different scores of ALDH protein expression. In particular, in case 1, we noted a low ALDH expression (score 1). This patient had a very good outcome: he had recurrence, but achieved remission of the disease after chemo- and radiotherapeutic treatments. In case 2, the patient showed a higher ALDH expression (score 3), and the patient died due to disease recurrence. This may indicate that the severity of the ALDH score could link with the worst prognosis and viceversa. This is the first report showing the presence of the ALDH marker in angiosarcoma of the lung. Notably, this is a report of only two cases; however, although the publications in this field are limited, we believe that the current data on this marker in this disease support future studies in a larger cohort of patients to test the presence of this marker and, possibly, determine whether prognosis is correlated with the expression of this protein. If the results from this report will be confirmed in a larger population, it will be interesting to consider new targeted treatment based on ALDH, which is currently being tested in a clinical trial for hematological malignancies (9).

### Limitations

Our study is the first report showing the presence of ALDH1A1 in lung angiosarcoma. We are conscious that this is a brief report of only two clinical cases, probably due to the rarity of this disease. Further multicentric studies will need to be set to confirm our data. However, we believe that our report is important to amplify the knowledge regarding possible markers which have been already studied in non-small cell lung cancer showing a high impact of this marker on the prognosis, as well as also in responses to cancer treatments (9, 10, 13, 33, 34).

In conclusion, although not confirmed in a larger cohort of patients for lung angiosarcoma, ALDH targeted therapy may be an effective treatment perspective for this malignancy, which usually appears at a high grade with an aggressive prognosis.

## Data Availability Statement

The raw data supporting the conclusions of this article will be made available by the authors, without undue reservation.

## Ethics Statement

The studies involving human participants were reviewed and approved by Ethics committee at University Hospital of Modena, MODENA, Italy, on 4 June 2019, Prot. N. 395/2019/OSS/AOUMO. Consent for publication of data has been obtained from study participants.

## Author Contributions

The idea for the manuscript was conceived in February 2019 by BA and was further developed by VM, DB, BM, FB, RD'A, FB, MD, UM, and AM was involved in histopathological diagnosis. BA and VM wrote the first draft of the manuscript. BA and UM had been involved in surgery and tissue collection. VM performed laboratory experiments. BA, VM, FB, MD, RD'A, AM, and UM all reviewed the manuscript and were involved in its critical revision before submission. All authors read and approved the final manuscript.

## Conflict of Interest

The authors declare that the research was conducted in the absence of any commercial or financial relationships that could be construed as a potential conflict of interest.

## Supplementary Material

Supplementary Figure 1 Immunohistochemical analysis of anti CD34 and CD31 in angiosarcoma of the lung. Paraffin-embedded tissues were stained with anti-CD34 and anti-CD31 antibodies used for the diagnosis of the angiosarcoma of the lung. **(A–C)** Representative immunohistochemical staining of anti-CD34. **(D–F)** Representative immunohistochemical staining of anti-CD31. Representative images are shown, with 10 × and 20 × magnification. Scale bar = 100 μm.

## Abbreviations

ALDH: aldehyde dehydrogenase
CT: computed tomography
DAB: 3,3-diaminobenzidine
DIMATE: dimethyl ampal thiolester
PBS: phosphate buffered saline
VATS: video-assisted thoracic surgery
^18^F-FDG PET/CT: 18-fluorodeoxyglucose integrated with computed tomography.
