# Supplementary figures and images for: ALDH Expression in Angiosarcoma of the Lung: A Potential Marker of Aggressiveness?
Source: Front Med (Lausanne). 2020 Oct 30;7:544158. doi: 10.3389/fmed.2020.544158 (PMC7662079; doi:10.3389/fmed.2020.544158)

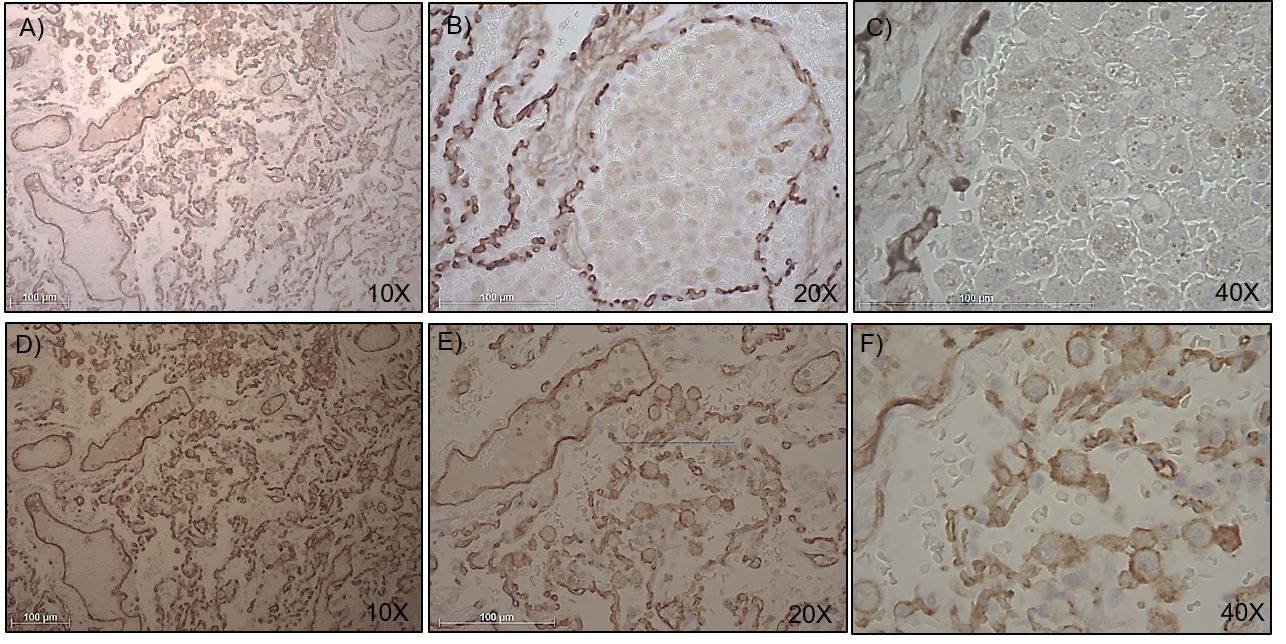

Supplement: Supplementary Figure 1 — Immunohistochemical analysis of anti CD34 and CD31 in angiosarcoma of the lung. Paraffin-embedded tissues were stained with anti-CD34 and anti-CD31 antibodies used for the diagnosis of the angiosarcoma of the lung. (A–C) Representative immunohistochemical staining of anti-CD34. (D–F) Representative immunohistochemical staining of anti-CD31. Representative images are shown, with 10 × and 20 × magnification. Scale bar = 100 μm. [file Image_1.TIF]
